# Replication-incompetent gammaretroviral and lentiviral vector-based insertional mutagenesis screens identify prostate cancer progression genes

**DOI:** 10.18632/oncotarget.24503

**Published:** 2018-02-15

**Authors:** Victor M. Bii, Casey P. Collins, Jonah D. Hocum, Grant D. Trobridge

**Affiliations:** ^1^ College of Pharmacy, Washington State University, Spokane 99210, WA, USA; ^2^ School of Molecular Biosciences, Washington State University, Pullman 99164, WA, USA

**Keywords:** prostate cancer (PC), insertional mutagenesis screen, gammaretroviral (γRV) vector, lentiviral (LV) vector, driver genes, Chromosome

## Abstract

Replication-incompetent gammaretroviral (γRV) and lentiviral (LV) vectors have both been used in insertional mutagenesis screens to identify cancer drivers. In this approach the vectors stably integrate in the host cell genome and induce cancers by dysregulating nearby genes. The cells that contain a retroviral vector provirus in or near a proto-oncogene or tumor suppressor are preferentially enriched in a tumor. γRV and LV vectors have different integration profiles and genotoxic potential, making them potentially complementary tools for insertional mutagenesis screens. We performed screens using both γRV and LV vectors to identify driver genes that mediate progression of androgen-independent prostate cancer (AIPC) using a xenotransplant mouse model. Vector transduced LNCaP cells were injected orthotopically into the prostate gland of immunodeficient mice. Mice that developed tumors were castrated to create an androgen-deficient environment and metastatic tumors that developed were analyzed. A high-throughput modified genomic sequencing PCR (MGS-PCR) approach identified the positions of vector integrations in these metastatic tumors. *OR2A14*, *FER1L6*, *TAOK3*, *MAN1A2*, *MBNL2*, *SERBP1*, *PLEKHA2*, *SPTAN1*, *ADAMTS1*, *SLC30A5*, *ABCC1*, *SLC7A1* and *SLC25A24* were identified as candidate prostate cancer (PC) progression genes. *TAOK3* and *ABCC1* expression in PC patients predicted the risk of recurrence after androgen deprivation therapy. Our data shows that γRV and LV vectors are complementary approaches to identify cancer driver genes which may be promising potential biomarkers and therapeutic targets.

## INTRODUCTION

Replication-incompetent retroviral vectors have the ability to stably integrate into the host cell genome and dysregulate nearby proto-oncogenes or tumor suppressor genes, ultimately leading to vector-induced cancer. Vector-mediated genotoxicity was unfortunately observed in hematopoietic stem cell (HSC) gene therapy clinical trials for X-linked severe combined immunodeficiency disease [1, 2], where integrations near or in the oncogene LIM domain only 2 (*LMO2*) resulted in increased expression via enhancer activation [3, 4]. Although other vector-mediated mechanisms of genotoxicity have been reported [5, 6], enhancer-mediated activation of gene promoters is the most common [7, 8]. Following these studies replication-incompetent retroviral vectors have been developed as a powerful tool to identify driver genes [9–14].

Replication-incompetent retroviral vectors have several advantages over traditionally used replicating retroviruses and transposons [9–13]. Replicating retroviruses, such as murine leukemia virus and mouse mammary tumor virus, generate secondary integrations which can make it difficult to distinguish driver genes from passenger genes [15]. Transposon mutagenesis screens in transgenic mouse models have been performed but their application in human systems are currently limited due to lack of efficient transposition [16]. Transposons are also subject to re-integration and local hopping events in the host genome, which can also complicate identification of driver genes. Replication-incompetent retroviral vectors have the ability to stably infect many mammalian cell types without causing secondary integrations that may impede the identification of the primary retroviral integration sites (RISs). This makes them a powerful tool to identify driver genes in different human cancers [14].

The two most commonly used replication-incompetent vectors for mutagenesis screens, gammaretroviral (γRV) and lentiviral (LV) vectors, have distinct integration profiles. γRV vectors have a high propensity to integrate near expressed gene regions, transcription start sites, and gene promoters [17]. LV vectors integrate preferentially into transcriptionally active gene regions [18]. γRV are also more genotoxic than LV vectors, with a higher likelihood of inserting near proto-oncogenes [8]. In addition to their unique integration profiles, the retroviral vector backbone design also influences genotoxicity. For example, retroviral vectors that contain enhancers in their long terminal repeats (LTRs) are more genotoxic than those with self-inactivating (SIN) LTRs, which have enhancers deleted from the U3 region, thereby limiting transcription to an internal promoter [19]. The type of internal promoter used can also influence genotoxicity. Strong viral promoters, such as the spleen focus forming virus (SFFV) promoter, have been shown to dysregulate nearby genes at a higher frequency than weak housekeeping promoters, such as the phosphoglycerate kinase promoter [7, 8].

The analysis of RISs allows identification of the location of dysregulated candidate cancer driver genes in the genome. To map RISs in mutagenesis screens, PCR and non-PCR based approaches have been used to identify the junctions between the retroviral LTR and chromosomal DNA [10, 20–22]. Shuttle vector rescue, a non-PCR based approach, generates LTR-chromosome junctions that are typically longer than those produced by PCR and can improve the detection of RIS in repetitive regions, whereas modified genomic sequencing PCR (MGS-PCR) is more sensitive [14]. The sequence reads obtained from these approaches are mapped to the genome and nearby candidate driver genes can be rapidly identified using bioinformatics tools such the vector integration site analysis (VISA) webserver [23]. In our previous study using a γRV shuttle vector insertional mutagenesis screen, we identified *SHARPIN* as a breast cancer metastasis gene that predicts metastasis-free survival in patients post-treatment [9]. In prostate cancer (PC) studies using LV shuttle vector rescue and MGS-PCR based approaches androgen-independent PC (AIPC) progression genes were identified [10, 11]. Other LV vector-based mutagenesis screens have used linear amplified mediated PCR (LAM-PCR) to identify driver genes in liver cancer [12] and drug resistance driver genes in breast and pancreatic cancers [13].

Here we directly compare the ability of both γRV and LV vectors to identify driver genes that mediate progression of AIPC in an *in vivo* mouse model. We hypothesized that the γRV and LV vectors would likely dysregulate different genes and might be used to identify candidate genes that would otherwise be missed when only one vector type is used. We identified several candidate PC progression genes including *TAOK3* and *ABCC1*. Analysis of PC patient data showed that *TAOK3* and *ABCC1* expression predicted disease recurrence-free survival in patients after treatment.

## RESULTS

### γRV and LV vector design and the generation of vector mutagenized PC cells

**Figure 1:**
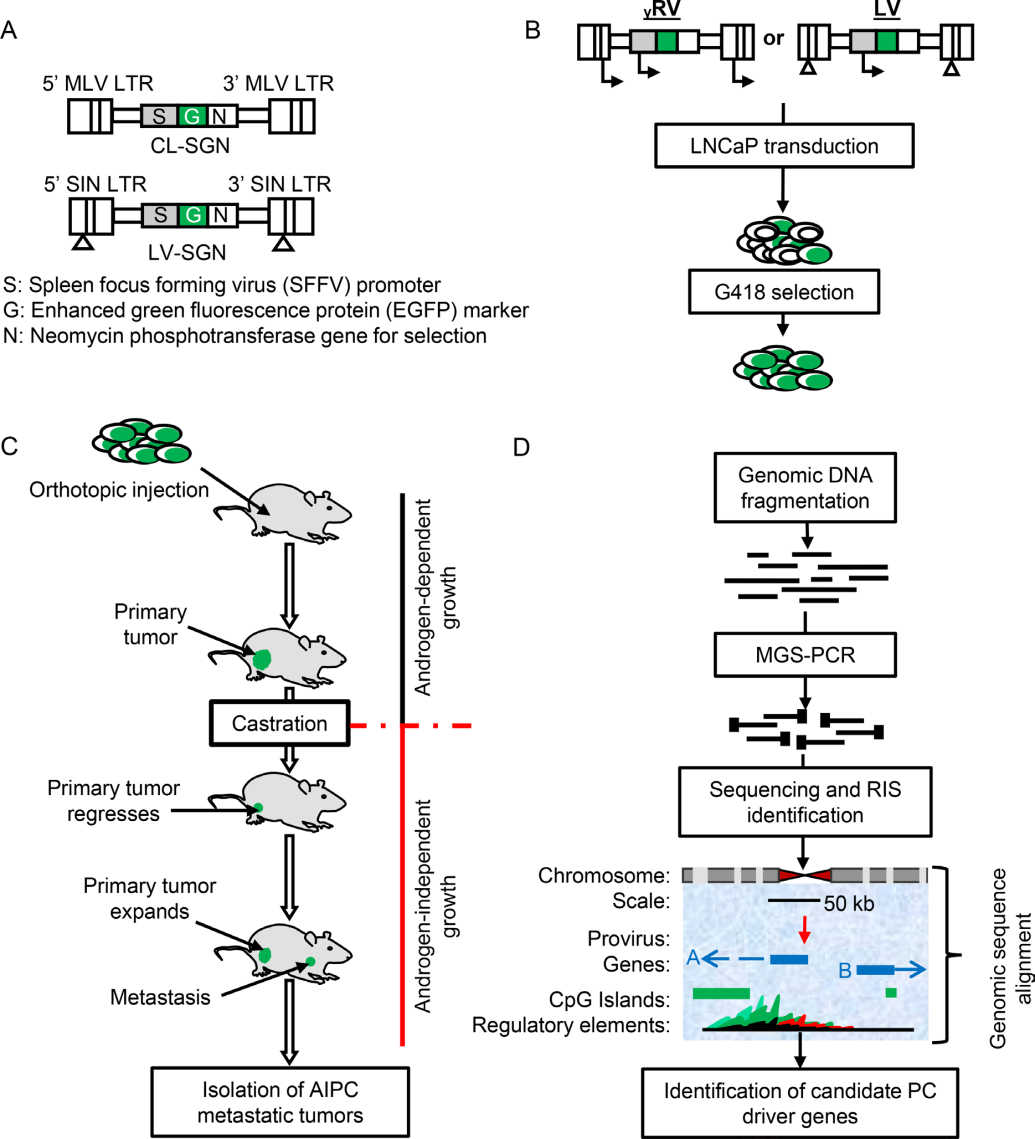
Retroviral vectors and insertional mutagenesis screen outline (**A**) Schematic representation of γRV and LV vector constructs. (**B**) Retroviral transduction and selection of transduced cells. (**C**) The androgen-independent xenotransplant mouse model. (**D**) The application of modified genomic sequencing PCR (MGS-PCR) to identify retroviral integration sites (RISs) from genomic DNA obtained from androgen-independent prostate cancer (AIPC) metastatic tumors.

**Figure 2:**
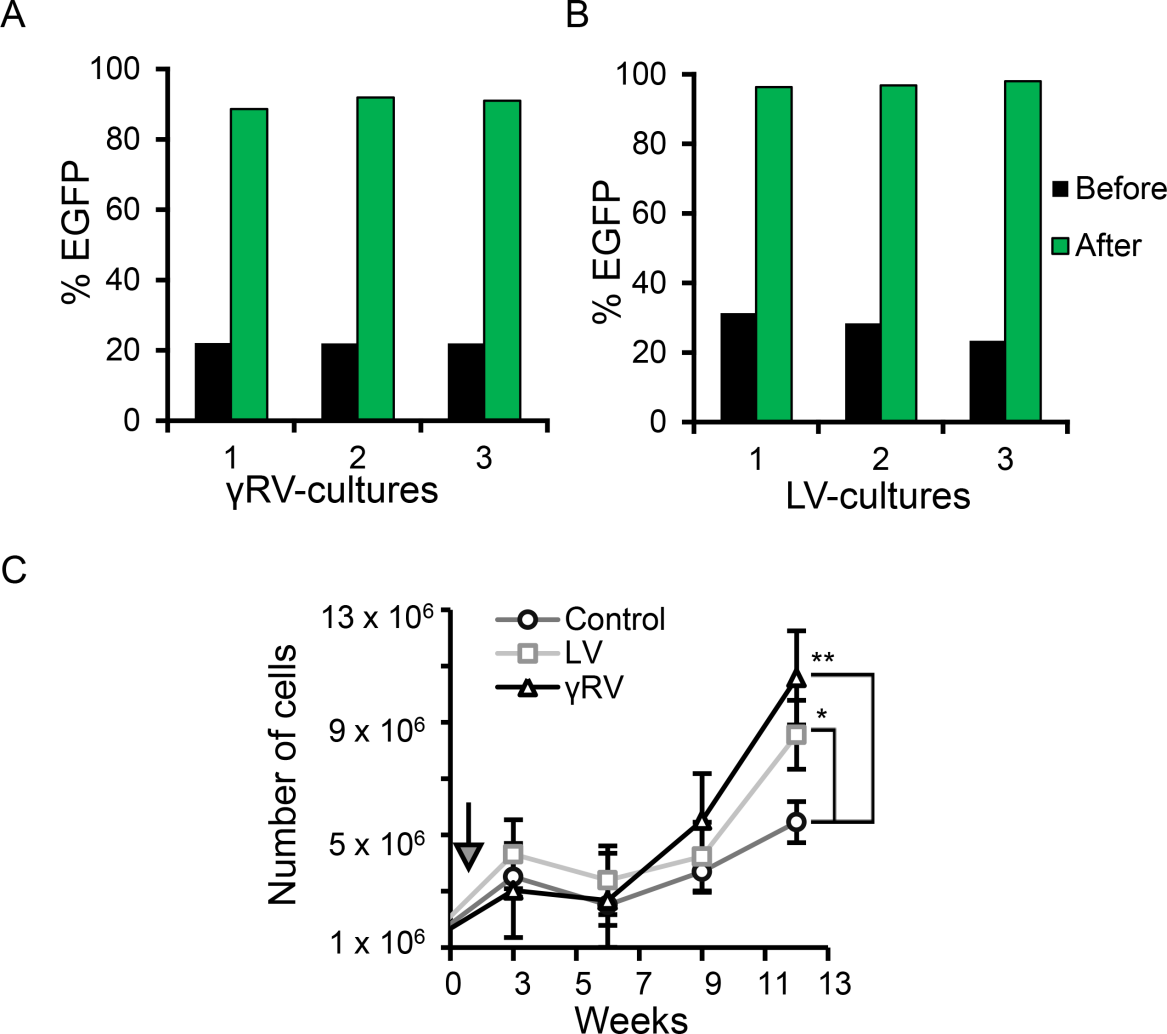
*In vitro* culture of PC cells in androgen-deficient condition G418 selection of (**A**) γRV and (**B**) LV vector transduced LNCaP cultures. (**C**) LNCaP cell proliferation in androgen-deficient conditions (*t* = 12 weeks). Arrow indicates the start of culturing cells in 100% CTS-FBS treated media (week 1). Data are the mean, error bars represents the SEM (^**^p < 0.01, ^*^p < 0.05).

The γRV and LV vectors are shown in Figure 1A. The γRV vector contains murine leukemia virus (MLV) LTRs with enhancer and promoter elements. The LV vector contains SIN LTRs that have the enhancer and promoter elements deleted in the LTR U3 region. Both vectors contain identical transgene cassettes of the SFFV promoter driving an enhanced green fluorescent protein (EGFP)-neomycin phosphotransferase fusion gene. High-titers of γRV and LV vectors (1.8 × 10^6^ TU/mL and 1.7 × 10^8^ TU/mL respectively) were produced and used to transduce human LNCaP cells at a multiplicity of infection (MOI) of 0.3 (Figure 1B). We used a relatively low MOI to limit the generation of clones containing multiple insertions [24]. Three independent parallel cultures were established for both γRV and LV vectors. The neomycin phosphotransferase gene allowed for efficient selection of transduced cells. All established cultures had > 90% transduced cells after G418 selection as assessed by flow cytometry for EGFP (Figure 2A and 2B). This approach of transducing at a low MOI followed by selection to remove untransduced cells minimizes multiple vector insertions that can make it difficult to identify driver genes from passengers [14].

### γRV and LV vector mutagenized PC cells induce tumor growth *in vivo*

**Figure 3:**
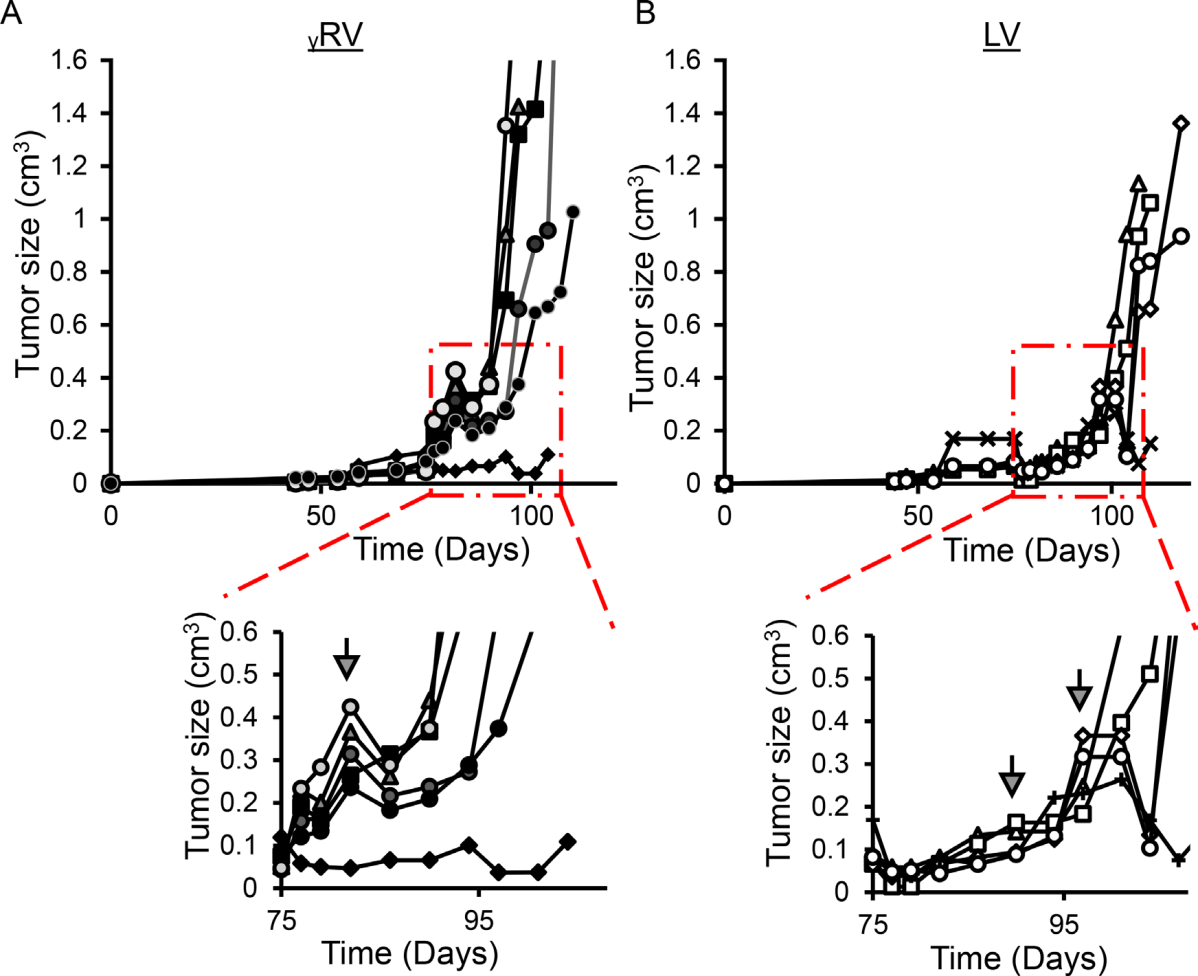
*In vivo* androgen-independent prostate cancer (AIPC) tumor growth (**A**) γRV vector derived AIPC primary tumors. (**B**) LV vector derived AIPC primary tumors. Mouse xenografts containing γRV vector transduced LNCaP cells established primary tumors earlier with a median castration point at 82 days compared with LV vector at 94 days (^**^p < 0.01). Arrows indicate castration time points.

To compare the ability of γRV and LV vector proviruses to induce androgen-independence, the androgen-dependent human PC LNCaP cell line was chosen due to its ability to develop androgen-independence when cultured in the absence of androgen [10, 25, 26]. Transduced and control non-transduced LNCaP cells were cultured in media with serum treated with charcoal. Charcoal removes steroid hormones from the media including androgen, and models *in vitro* the androgen-deficient conditions in PC patients after androgen deprivation therapy. The LNCaP cells were cultured in 97.5% charcoal dextran-treated fetal bovine serum (CT-FBS) supplemented with 2.5% fetal bovine serum (FBS) for one week to minimize the loss of cells which would reduce the clonality of our mutagenized library. The cells were then cultured in 100% CT-FBS which initially resulted in reduced cell numbers as expected. The elimination of androgen may have resulted in apoptosis of some cells between weeks three and six (Figure 2C). As expected, LNCaP cultures became androgen-independent more rapidly in both the γRV and LV vector mutagenized cells compared to the non-transduced controls (*p*-value < 0.001 and 0.05 respectively) (Figure 2C). In order to directly compare the ability of γRV and LV vectors to induce androgen-independent growth *in vivo,* we used a previously described mouse xenograft model for AIPC (Figure 1C) [11, 27]. Nine out of 12 mice injected orthotopically in the prostate with transduced cells efficiently developed primary tumors post-injection (γRV, *n* = 5 and LV, *n* = 4) (Figure 3). One mouse injected with LV vector died following injection. Mouse xenografts containing γRV vector transduced LNCaP cells established primary tumors earlier with a median of 82 days compared with LV vector at 94 days (*p* < 0.001) (Figure 3). The mice were castrated when the primary tumor sizes reached a volume of approximately 0.2 cm^3^. The tumors regressed for approximately 1 week post-castration as measured by tumor volumes. After 2–3 week of the initial regression all the animals showed enhanced tumor growth which models what is observed in human PC patients after androgen deprivation therapy. γRV vector derived tumors grew faster than LV tumors, but this trend was not statistically significant (*p* = 0.14) (Figure 3). At the end of the experiment 83% of animals that developed primary tumors had metastasis in lung, liver, kidney or spleen.

### MGS-PCR sequencing of retroviral integration sites in metastases

To identify the γRV and LV vector integration sites and nearby candidate genes that promote PC progression, we analyzed the lung and liver metastatic tumors obtained from castrated mice (Figure 1D). The genomic DNA was isolated and retroviral integrations were amplified using MGS-PCR as previously described [20, 21]. LTR-chromosomal junction sequence reads were mapped to the human genome (hg38) using the VISA bioinformatics program and nearby candidate genes were identified [23]. We identified 40 and 76 unique RISs from γRV and LV vector derived lung and liver metastatic tumors respectively, that could be mapped to the human genome (Table 1 and Supplementary Figure 1). For each vector type, the frequency of provirus integration sites recovered were similar.

**Table 1:** MGS-PCR identify γRV and LV vector integration sites at similar frequency in AIPC metastases

	Provirus Integration Sites		
Retroviral vector	Lungs	Liver	Total
ᵧRV	31 (*n* = 3)	9 (*n* = 2)	40 (*n* = 5)
LV	58 (*n* = 3)	18(*n* = 2)	76 (*n* = 5)

n: Indicates total number of metastases analyzed.

We analyzed metastatic tumors obtained from lung and liver tissues within and between animals to determine if different or similar candidate genes identified were responsible for driving tumor progression. For example, LV vector-derived liver and lung metastasis obtained from a single animal identified *TBC1D5*, *MAN1A1*, *UQCRC1*, *ABBC1*, *DAP3*, and *VPS13D* candidate genes in both tissues, whereas *TP53*, *MAN1A2*, *PPMIE*, *PLEKHA2*, *PRKCA*, *PDXDC2P*, *ENG*, *PPP4R2*, *XRN1*, *DAOA-AS1*, and *LOC101060091* were identified only in lung tissue. Analysis of metastases from two animals that received cells from the same LV vector transduction identified similar candidate genes in lung and liver metastasis including *ABCC1*, a candidate progression gene (see below) which was present in three metastases. Genes with a vector provirus less than 50 kb from the TSS or within the transcription unit were considered for further analysis (Supplementary Table 1) resulting in 58 potential candidate genes.

### Meta-analysis of candidate PC progression genes identified by γRV and LV vectors

We combined the data from our screen with publicly available Oncomine^™^ microarray gene expression data [28] from PC patient tumor samples to identify candidate genes. We reasoned that combining patient data with the candidate genes identified from our screen should improve the ability to identify genes that are clinically relevant. Oncomine^™^ meta-analysis of 21 independent datasets [29–44] were used to evaluate the 58 candidate genes. 35 were overexpressed, 18 were underexpressed while 5 had no expression data available (Supplementary Table 1). Out of 31 candidate genes with LV vector integrations, 15 were overexpressed, 11 were underexpressed, and 5 genes did not have any expression data available. For the 27 candidate genes with γRV integrations, 20 were overexpressed and 7 were underexpressed. Of these genes, 13 had significant differences in expression levels (*p* < 0.05) by meta-analysis and were considered for further analysis (Table 2). Of the selected candidate genes, γRV vector integrations identified *OR2A14*, *FER1L6*, *TAOK3*, *MBNL2*, *SERBP1*, *SLC7A1*, and *SLC25A24* while LV vector integrations identified *MAN1A2*, *PLEKHA2*, *SPTAN1*, *ADAMTS1*, *SLC30A5*, and *ABCC1*. We selected γRV vector-tagged *TAOK3* and LV vector-tagged *ABCC1* as our top candidate genes for further analysis. Both genes were overexpressed in PC patient samples available in 52% (11/21) of the datasets. *TAOK3* and *ABCC1* overexpression appear to affect known biological processes that may influence AIPC progression. *TAOK3* overexpression activates mitogen-activated protein kinases (MAPK), a known cancer signaling pathway, via ERK1/ERK2, JNK/SAPK and p38 [45, 46]. *TAOK3* is also involved in hepatocellular carcinoma and has been shown to be an androgen-responsive gene [47, 48]. *ABCC1* is overexpressed in small cell lung carcinoma metastases at relapse after chemotherapy [49] and is an established member of the ATP-binding cassette (ABC) transport protein family, shown to influence chemoresistance in brain, breast, liver, and prostate cancers [50–53].

**Table 2:** Candidate AIPC progression genes

Chr.^1^	Gene^2^	In gene^3^	Distance from TSS^4^	Up or downstream^5^	Expression^6^	*P*-value^7^	Vector^8^	Tissue^9^
7	*OR2A14*	No	3546	Downstream	Over	0.000145	γRV	Lung
8	*FER1L6*	No	11278	Upstream	Over	0.000242	γRV	Liver
12	***TAOK3***	Yes	9210	Downstream	Over	0.000657	γRV	Lung
1	*MAN1A2*	Yes	53962	Downstream	Under	0.002	LV	Lung, Liver
13	*MBNL2*	Yes	134465	Downstream	Under	0.002	γRV	Lung
1	*SERBP1*	Yes	180	Downstream	Over	0.008	γRV	Lung
8	*PLEKHA2*	Yes	53671	Downstream	Under	0.011	LV	Lung
9	*SPTAN1*	Yes	42111	Downstream	Under	0.015	LV	Lung
21	*ADAMTS1*	No	19479	Upstream	Under	0.021	LV	Lung
5	*SLC30A5*	No	16882	Upstream	Over	0.03	LV	Lung
16	***ABCC1***	Yes	181992	Downstream	Over	0.031	LV	Lung, Liver
13	*SLC7A1*	Yes	71506	Downstream	Over	0.046	γRV	Liver
1	*SLC25A24*	Yes	44414	Downstream	Under	0.049	γRV	Lung

1. Chromosome with vector provirus.

2. Gene tagged by vector provirus.

3. Indicates if the vector provirus is within the gene.

4. Vector provirus distance to the transcription start site (TSS).

5. Indicates if the vector provirus is upstream or downstream from the TSS.

6. Expression of the candidate gene in PC patient tissue from Oncomine™ analysis.

7. p-value from Oncomine™ analysis of expression between PC patient tissue and unaffected tissue.

8. Vector type used for insertional mutagenesis.

9. Tissue from which the metastasis was isolated.

### *TAOK3* and *ABCC1* are recurrently altered in PC patients and have prognostic value

**Figure 4:**
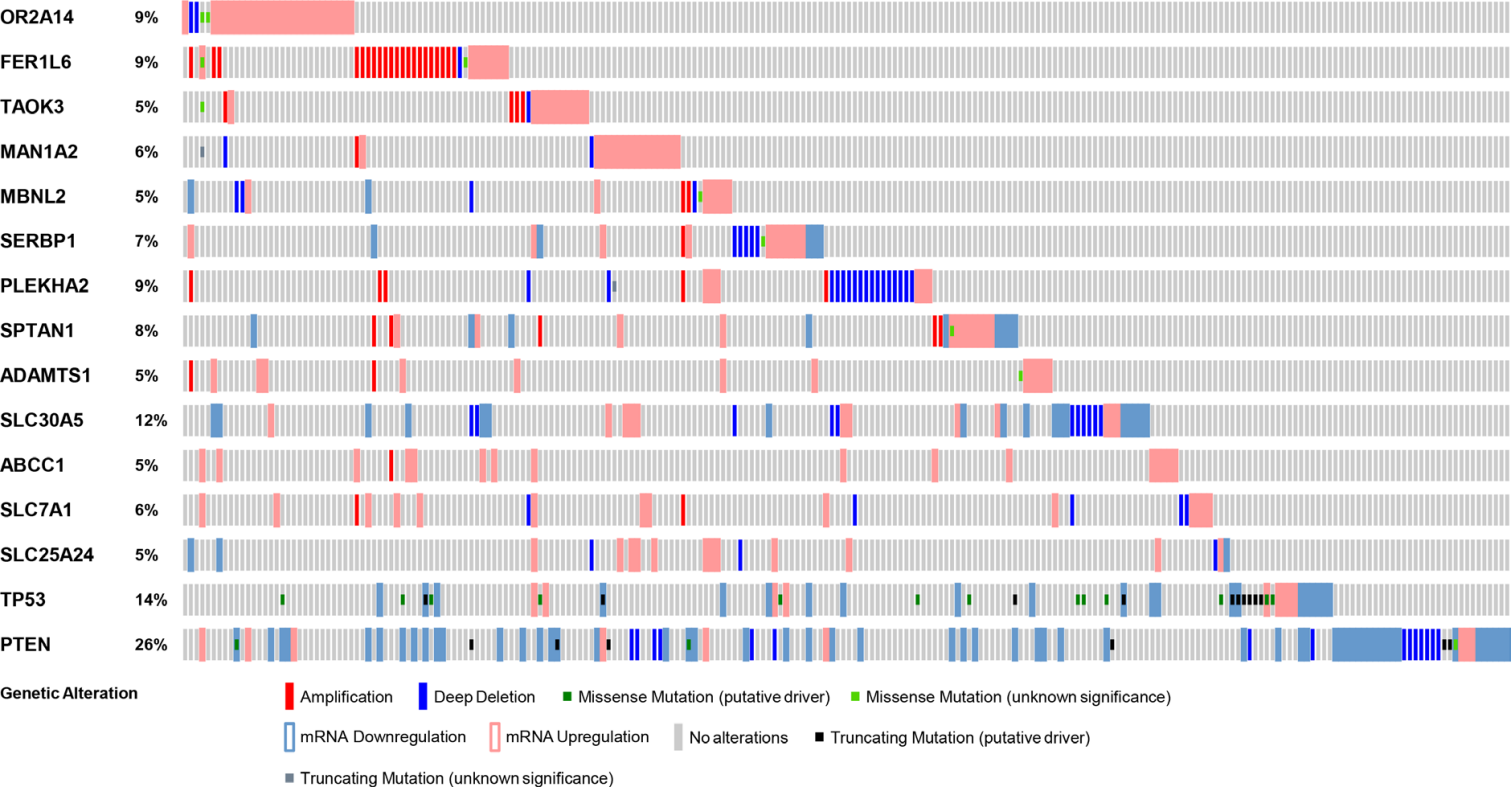
Genetic alterations in *TAOK3* and *ABCC1* genes in PC patients Each patient sample is represented by a bar and each color indicates specific genetic alteration as indicated. Only patients with alterations were shown (214/333). As controls, genetic alteration of *TP53* and *PTEN*, frequently altered genes in PC patients is also shown. The frequency of gene alteration is represented as a percentage.

**Figure 5:**
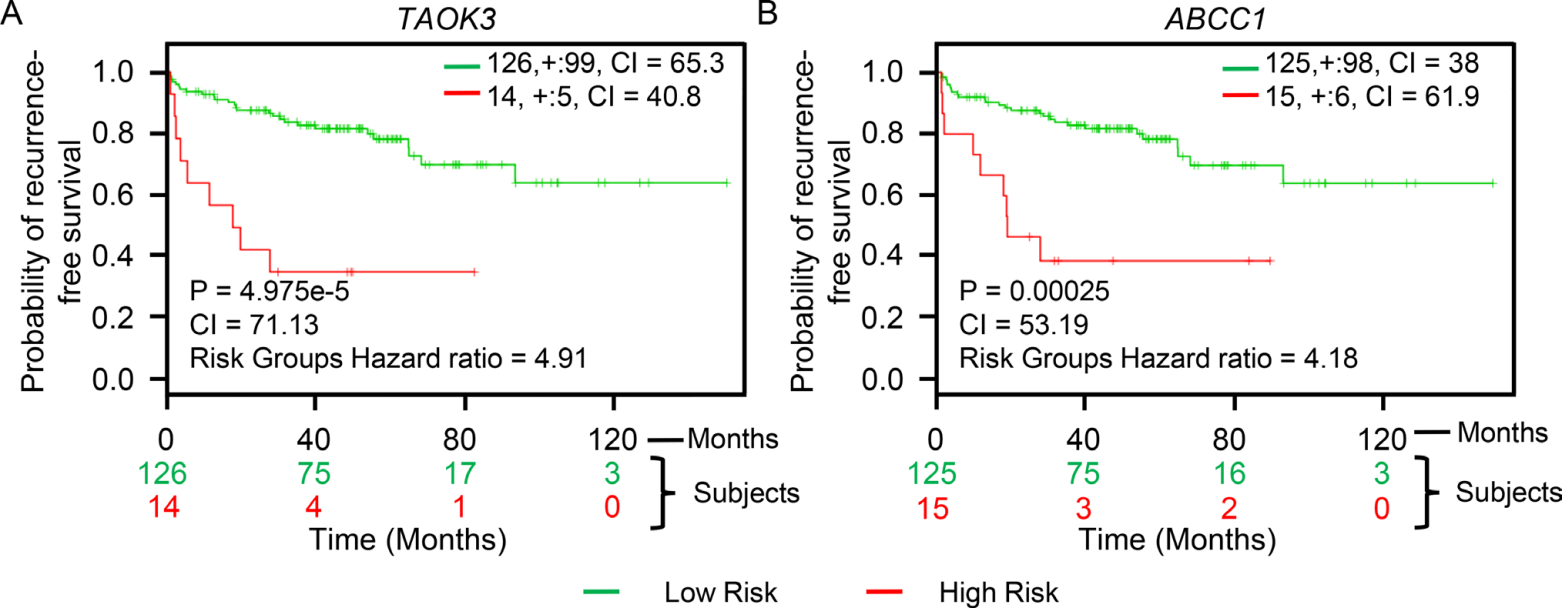
*TAOK3* and *ABCC1* expression predicts recurrence-free survival in AIPC patients (**A**) *TAOK3.* (**B**) *ABCC1.* The Kaplan–Meier survival curves generated using the SurvExpress biomarker tool shows the ability of retroviral-tagged gene expression to predict recurrence-free survival outcome in PC patients after androgen deprivation therapy. The insets in top right represents number of individuals, number censored, and concordance index (CI) of each risk groups and ‘+’ represent censoring samples. High and low risk groups are shown in red and green respectively. Box-plots show expression levels and *p*-values resulting from *t*-test of the difference expression between high risk (red) and low risk (green) groups in PC patients.

Analysis of genes that are mutated can inform predictions of patient tumor progression and assist with decisions concerning treatment options [9, 54]. Apart from a few studies, such genome-wide profiling in PC patients has been limited [38, 55]. Genomic alterations, including mutations and gene copy number variations, have been shown to affect important PC pathways [56]. Thus, the frequency of genetic alterations in PC patients for the genes we have identified, including *TAOK3* and *ABCC1*, may provide important information for PC progression. We utilized the cBioPortal tool to evaluate different genetic alterations including DNA copy number alterations (deletions and amplifications) [57] in the TCGA dataset [58] to determine if they relate to PC progression. The TCGA dataset was used because of its large sample size (333 patient tumor samples) and because it included all candidate PC progression genes that we identified. For comparison, we also evaluated *TP53* and *PTEN*, two mutated tumor suppressors found altered at high frequency in PC patients. The frequency of alteration for both *TAOK3* and *ABCC1* in PC patient samples was 5% (Figure 4). Other candidate genes we identified had frequencies between 5% and 12% (Figure 4). We next assessed whether the expression of *TAOK3* and *ABCC1* predicted clinical outcome using patient data associated with long-term clinical follow-up. Using the publicly available SurvExpress biomarker tool [59] that stratifies PC patients into low or high-risk groups based on differential gene expression, we generated Kaplan–Meier survival curves using the data of Taylor *et al.* [38]. In this study 140 patients were stratified based on disease recurrence and staging after androgen deprivation therapy. Expression of *TAOK3* and *ABCC1* significantly reduced recurrence-free survival in patients (*TAOK3: p* = 4.975e-5, Concordance Index = 71.13, Risk Groups Hazard Ratio = 4.91 and *ABCC1: p* = 0.00025, Concordance Index = 53.19, Risk Groups Hazard Ratio = 4.18) (Figure 5). In addition, when a combination of *TAOK3* and *ABCC1* were used, the prediction of recurrence-free survival of PC patients using Taylor *et al.* data was significant (*p* = 0.00042, Concordance Index = 70.75, Risk Groups Hazard Ratio = 3.91) (Supplementary Figure 2). This shows that *TAOK3* and *ABCC1* expression is a prognostic indicator for survival in AIPC patients after androgen deprivation therapy.

## DISCUSSION

In this study, we have shown that retroviral vector insertional mutagenesis screens using both γRV and LV vectors are powerful and complementary tools to identify genes that drive PC progression. Insertional mutagenesis screens using replication-incompetent retroviral vectors have been used previously to identify driver genes in prostate [10, 11], liver [12], pancreatic [13] and breast [9, 13] cancers. However, our study is unique because it is the first to our knowledge to directly compare insertional mutagenesis screens using both γRV and LV vectors to identify driver genes. Currently, PC is the second leading cause of cancer-related death in American men and androgen deprivation therapy is the primary line of treatment [60]. However, the development of metastatic AIPC [61] is the main cause of mortality for PC patients [62] and the molecular processes of AIPC progression are poorly understood. We identified *OR2A14*, *FER1L6*, *TAOK3*, *MAN1A2, MBNL2*, *SERBP1*, *PLEKHA2*, *SPTAN1*, *ADAMTS1*, *SLC30A5*, *ABCC1*, *SLC7A1* and *SLC25A24* as candidate genes implicated in PC progression. The frequency of identified genes was similar for γRV (7) and LV (6) vectors (Table 2).

In some cases different metastatic tumors from the same animal, such as liver or lung, contained proviral insertions in different genomic loci with different potential driver genes, supporting previous studies that individual metastases may be biologically heterogeneous, complicating potential treatment options [55, 63]. Also, some metastatic tumors from tissues obtained from the same animal or different animals had similar provirus insertions in the same loci tagging similar candidate genes, supporting previous studies that similar mutations can drive tumorigenesis in and between PC patients [38]. These observations may provide a rationale for developing future combinatorial or individualized treatment options for PC patients to overcome resistance to primary androgen deprivation therapy.

The identification of candidate genes that are altered during PC progression can reveal potential novel therapeutic targets and biomarkers. Analysis of patient data showed that *TAOK3* and A*BCC1* are significantly overexpressed in tumors compared to normal tissues (Table 2). PC patients had frequent genetic alterations in *TAOK3* and *ABCC1* including amplification, mRNA upregulation, and deletion, further suggesting their involvement in tumorigenesis (Figure 4). We also found association of *TAOK3* and *ABCC1* expression levels with PC patient clinical outcome. The expression of *TAOK3* and *ABCC1* singly or in combination predicted recurrence-free survival after androgen deprivation therapy using the Taylor *et al.* PC patient dataset [38]. Thus *TAOK3* and *ABCC1* are important AIPC prognostic biomarkers that can determine the potential of disease progression and stratify patients according to treatment needs.

Previous studies have shown that *TAOK3* and *ABCC1* are involved in various cancers [47, 48, 50–53]. The overexpression of *TAOK3* has been shown to activate known cancer pathways that regulate cell survival, growth and differentiation [45, 46]. *ABCC1* has been shown to regulate chemoresistance in glioma, breast, prostate, and liver cancers [50–53]. It is likely that known cancer signaling pathways reported for these genes in other cancer types might be implicated in AIPC progression.

In conclusion, the combined use of γRV and LV mutagenesis screens identified *TAOK3* and *ABCC1* as potential PC driver genes that can predict disease recurrence in patients with AIPC. Combining the use of γRV and LV vectors is a powerful approach to identify genes involved in oncogenic processes with broad potential application for numerous clinically relevant oncogenic processes in diverse cancer types.

## MATERIALS AND METHODS

### Cell line, vector production, and transduction

The androgen-dependent human prostate carcinoma cell line LNCaP-FGC (ATCC CRL-1740, Rockville, MD, USA) was cultured in RPMI-1640 (Thermo Scientific, Waltham, MA, USA) supplemented with 10% FBS (Atlanta Biologicals, Lawrenceville, GA, USA) and penicillin/streptomycin at 37° C in 5% CO_2_. The γRV vector, CL-SGN, was previously described [9]. The LV vector, LV-SGN, has SIN LTRs, a strong internal SFFV promoter driving an EGFP-neomycin fusion protein. Concentrated γRV and LV vector stocks pseudotyped with a vesicular stomatitis virus-glycoprotein (VSV-G) envelope were produced by polyethylenimine transient transfection of human embryonic kidney 293 (HEK 293T) cells using helper plasmids pLGPS (γRV vector), psPAX2 (LV vector) and the VSV-G envelope helper plasmid pMD2.G. Viral supernatant was filtered using 0.45 µm (γRV vector) and 0.22 µm (LV vector) filters (Pall Life Sciences, Cornwall, UK) and centrifuged for 18 h at 12,100 g. The viral supernatant was concentrated 100 fold. Viral titers were determined by transduction of HT1080 fibrosarcoma cells and analyzed for EGFP expression by flow cytometry.

### Generation of mutagenized human LNCaP cells

LNCaP cells were transduced at a MOI of 0.3 with either γRV or LV vectors and cultured in RPMI supplemented with 10% FBS. Cells were passaged and re-plated 1:2 every 3–4 days while under G418 selection (600 µg/ml) for 3 weeks.

### *In vitro* cultures of LNCaP cells in androgen-deficient conditions

Control, γRV and LV vector transduced LNCaP cells were maintained in RPMI-1640 supplemented with 10% CT-FBS (Atlanta Biologicals, Lawrenceville, GA, USA) media to remove steroid hormones. To minimize the loss of cells which would reduce clonality of our mutagenized library of RIS, we initially cultured cells for one week in 97.5% CT-FBS supplemented with 2.5% FBS [10]. After one week the *in vitro* cultures were then maintained in 100% CT-FBS treated media. Cell cultures for each group were established in triplicate. Cells were counted and 1 × 10^6^ cells passaged as described [10] to generate growth curves.

### *In vivo* PC metastasis model

All procedures involving handling of animals were performed according to protocols approved by the Washington State University Institutional Animal Care and Use Committee for human use of animals in research. 5–8 week old male NOD.Cg-Prkdc^scid^Il2rg^tmlWjl^/SzJ (NSG) mice were obtained from the Jackson Laboratory (Bar Harbor, ME, USA). γRV and LV vector LNCaP cells were mutagenized with three independent transductions at a MOI of 0.3 and were transplanted orthotopically into the dorsal prostate of 8 week old male mice (two animals per transduction). A 1 cm incision was made in the skin and peritoneum to expose the prostate gland. 1 × 10^6^ LNCaP cells mutagenized with either γRV or LV vector were suspended in 20 µl phosphate buffered saline (PBS) (Lonza, Walkersville, MD, USA) and injected using a 27 gauge needle into the dorsal prostate gland. The incision in the skin was closed using wound clips. Mice were monitored daily for 3 days after surgery, then every 3 days over the course of the experiment. The growth of primary tumors was determined twice weekly by external Vernier caliper measurement and volume of the primary tumor calculated using (Lwh) x 0.5236 as described [10]. The values were extrapolated and primary tumor growth curves were generated. *In vivo* androgen-independent tumor growth was established by castrating the mice when the primary tumor size reached approximately 0.2 cm^3^ via the scrotal approach. When the primary tumors reached sizes larger than the initial volumes prior to castration, the mice were euthanized. At necropsy, primary tumors and EGFP-positive metastases were harvested, snap-frozen in liquid nitrogen for approximately 10 seconds, and stored at −80° C. The genomic DNA was extracted from the metastatic tissues using a Qiagen PureGene Cell and Tissue Kit (Valencia, CA, USA).

### Identification of proviral integration sites

The genomic DNA was randomly sheared using a Hydroshear DNA shearing device (Digilab, Marlborough, MA, USA). The integration sites were identified using a previously described high-throughput MGS-PCR approach [20, 21]. Sequence reads ranging from 1.3 to 2.5 million were obtained per metastatic tumor. Forward and reverse sequence reads were paired to extend sequence read lengths using Paired-End reAd mergeR (PEAR) sequencing software [64]. The integrated provirus LTR-chromosomal junctions were identified and the genomic sequence mapped to the human genome (hg38) using VISA [23] and the University of California Santa Cruz BLAST-like alignment tool (UCSC BLAT) [65] (Supplementary Figure 1). A custom PERL programming tool was used to identify genes within 50 kb of the closest transcription start sites of nearby genes. Alignments that had canonical LTR-chromosomal junctions that met criteria as described [66] were considered as RIS.

### Identification and analysis of candidate PC metastasis genes

Publicly available cDNA microarray datasets in Oncomine^™^ database (Supplementary Figure 3) were used to assess and analyze gene expression in normal prostate versus PC patient tissues from the same patient tissue type [28]. The *p*-values for gene expression between the two classes was generated by Oncomine^™^ using Student’s *t*-test. Pre-computated differential gene expression profiles of candidate genes in each dataset served as an input for meta-analysis. 21 patient-derived microarray datasets [29–44] were used to independently evaluate expression patterns of the γRV and LV vector-tagged candidate metastasis genes with integrated proviruses (Supplemental Figure 3). A total of 1,010 PC samples and 498 normal samples were used. The online cBioPortal cancer genomics tool (http://www.cbioportal.org/) was used to assess the frequency of genetic alterations in patients expressing the candidate PC metastasis genes [57, 67]. TCGA datasets from 333 patients tumor samples with sequencing and copy number alteration data was used to determine the genetic alteration frequency in patients expressing candidate genes identified in our screen [58]. The SurvExpress online biomarker tool [59] was used to predict the clinical outcome and prognostic value of PC metastasis genes. We searched for mRNA expression across eight available PC mRNA datasets using candidate genes as searching criteria and selected the Taylor *et al.* [38] patient microarray dataset GSE21032 that assessed the recurrence-free survival of 140 PC patients with a 5 year median clinical follow-up after androgen deprivation therapy. We obtained results using average score from probe sets and the default quantile-normalized format. We set the statistical analysis and graphical outputs using available datasets endpoints to obtain two maximized risk groups (low and high risk). Kaplan–Meier survival curves of censored Cox survival analysis was generated and log-rank statistical test performed with significance at a 95% confidence level.

### Statistical analysis

Statistical analysis comparing the growth of γRV and LV vector transduced LNCaP cells versus their respective non-transduced controls in *in vitro* androgen-deficient conditions, the rate of γRV and LV-derived tumor growth *in vivo*, was performed using the Student’s *t*-test. Values were expressed as means ± SEM. *p*-values of < 0.05 were considered significant.

## SUPPLEMENTARY MATERIALS FIGURES AND TABLE





## References

[1] Cavazzana-Calvo M, Hacein-Bey S, de Saint Basile G, Gross F, Yvon E, Nusbaum P, Selz F, Hue C, Certain S, Casanova JL, Bousso P, Deist FL, Fischer A (2000). Gene therapy of human severe combined immunodeficiency (SCID)-X1 disease. Science.

[2] Hacein-Bey-Abina S, Le Deist F, Carlier F, Bouneaud C, Hue C, De Villartay JP, Thrasher AJ, Wulffraat N, Sorensen R, Dupuis-Girod S, Fischer A, Davies EG, Kuis W (2002). Sustained correction of X-linked severe combined immunodeficiency by *ex vivo* gene therapy. N Engl J Med.

[3] Hacein-Bey-Abina S, Von Kalle C, Schmidt M, McCormack MP, Wulffraat N, Leboulch P, Lim A, Osborne CS, Pawliuk R, Morillon E, Sorensen R, Forster A, Fraser P (2003). LMO2-associated clonal T cell proliferation in two patients after gene therapy for SCID-X1. Science.

[4] Hacein-Bey-Abina S, Garrigue A, Wang GP, Soulier J, Lim A, Morillon E, Clappier E, Caccavelli L, Delabesse E, Beldjord K, Asnafi V, MacIntyre E, Dal Cortivo L (2008). Insertional oncogenesis in 4 patients after retrovirus-mediated gene therapy of SCID-X1. J Clin Invest.

[5] Uren AG, Kool J, Berns A, van Lohuizen M (2005). Retroviral insertional mutagenesis: past, present and future. Oncogene.

[6] Cesana D, Sgualdino J, Rudilosso L, Merella S, Naldini L, Montini E (2012). Whole transcriptome characterization of aberrant splicing events induced by lentiviral vector integrations. J Clin Invest.

[7] Cesana D, Ranzani M, Volpin M, Bartholomae C, Duros C, Artus A, Merella S, Benedicenti F, Sergi Sergi L, Sanvito F, Brombin C, Nonis A, Serio CD (2014). Uncovering and dissecting the genotoxicity of self-inactivating lentiviral vectors *in vivo*. Mol Ther.

[8] Modlich U, Navarro S, Zychlinski D, Maetzig T, Knoess S, Brugman MH, Schambach A, Charrier S, Galy A, Thrasher AJ, Bueren J, Baum C (2009). Insertional transformation of hematopoietic cells by self-inactivating lentiviral and gammaretroviral vectors. Mol Ther.

[9] Bii VM, Rae DT, Trobridge GD (2015). A novel gammaretroviral shuttle vector insertional mutagenesis screen identifies SHARPIN as a breast cancer metastasis gene and prognostic biomarker. Oncotarget.

[10] Schinke EN, Bii V, Nalla A, Rae DT, Tedrick L, Meadows GG, Trobridge GD (2014). A novel approach to identify driver genes involved in androgen-independent prostate cancer. Mol Cancer.

[11] Nalla AK, Williams TF, Collins CP, Rae DT, Trobridge GD (2016). Lentiviral vector-mediated insertional mutagenesis screen identifies genes that influence androgen independent prostate cancer progression and predict clinical outcome. Mol Carcinog.

[12] Ranzani M, Cesana D, Bartholomae CC, Sanvito F, Pala M, Benedicenti F, Gallina P, Sergi LS, Merella S, Bulfone A, Doglioni C, von Kalle C, Kim YJ (2013). Lentiviral vector-based insertional mutagenesis identifies genes associated with liver cancer. Nat Methods.

[13] Ranzani M, Annunziato S, Calabria A, Brasca S, Benedicenti F, Gallina P, Naldini L, Montini E (2014). Lentiviral vector-based insertional mutagenesis identifies genes involved in the resistance to targeted anticancer therapies. Mol Ther.

[14] Bii VM, Trobridge GD (2016). Identifying Cancer Driver Genes Using Replication-Incompetent Retroviral Vectors.

[15] Lund AH, Turner G, Trubetskoy A, Verhoeven E, Wientjens E, Hulsman D, Russell R, DePinho RA, Lenz J, van Lohuizen M (2002). Genome-wide retroviral insertional tagging of genes involved in cancer in Cdkn2a-deficient mice. Nat Genet.

[16] Mirzaei H, Sahebkar A, Jaafari MR, Hadjati J, Javanmard SH, Mirzaei HR, Salehi R (2016). PiggyBac as a novel vector in cancer gene therapy: current perspective. Cancer Gene Ther.

[17] Wu X, Li Y, Crise B, Burgess SM (2003). Transcription start regions in the human genome are favored targets for MLV integration. Science.

[18] Schroder AR, Shinn P, Chen H, Berry C, Ecker JR, Bushman F (2002). HIV-1 integration in the human genome favors active genes and local hotspots. Cell.

[19] Montini E, Cesana D, Schmidt M, Sanvito F, Bartholomae CC, Ranzani M, Benedicenti F, Sergi LS, Ambrosi A, Ponzoni M, Doglioni C, Di Serio C, von Kalle C, Naldini L (2009). The genotoxic potential of retroviral vectors is strongly modulated by vector design and integration site selection in a mouse model of HSC gene therapy. J Clin Invest.

[20] Beard BC, Adair JE, Trobridge GD, Kiem HP (2014). High-throughput genomic mapping of vector integration sites in gene therapy studies. Methods Mol Biol.

[21] Rae DT, Collins CP, Hocum JD, Browning DL, Trobridge GD (2015). Modified Genomic Sequencing PCR Using the MiSeq Platform to Identify Retroviral Integration Sites. Hum Gene Ther Methods.

[22] Schmidt M, Schwarzwaelder K, Bartholomae C, Zaoui K, Ball C, Pilz I, Braun S, Glimm H, von Kalle C (2007). High-resolution insertion-site analysis by linear amplification-mediated PCR (LAM-PCR). Nat Methods.

[23] Hocum JD, Battrell LR, Maynard R, Adair JE, Beard BC, Rawlings DJ, Kiem HP, Miller DG, Trobridge GD (2015). VISA - Vector Integration Site Analysis server: a web-based server to rapidly identify retroviral integration sites from next-generation sequencing. BMC Bioinformatics.

[24] Kustikova OS, Wahlers A, Kuhlcke K, Stahle B, Zander AR, Baum C, Fehse B (2003). Dose finding with retroviral vectors: correlation of retroviral vector copy numbers in single cells with gene transfer efficiency in a cell population. Blood.

[25] Pousette A, Carlstrom K, Henriksson P, Grande M, Stege R (1997). Use of a hormone-sensitive (LNCaP) and a hormone-resistant (LNCaP-r) cell line in prostate cancer research. Prostate.

[26] Lu S, Tsai SY, Tsai MJ (1999). Molecular mechanisms of androgen-independent growth of human prostate cancer LNCaP-AI cells. Endocrinology.

[27] Wang X, An Z, Geller J, Hoffman RM (1999). High-malignancy orthotopic nude mouse model of human prostate cancer LNCaP. Prostate.

[28] Rhodes DR, Kalyana-Sundaram S, Mahavisno V, Varambally R, Yu J, Briggs BB, Barrette TR, Anstet MJ, Kincead-Beal C, Kulkarni P, Varambally S, Ghosh D, Chinnaiyan AM (2007). Oncomine 3.0: genes, pathways, and networks in a collection of 18,000 cancer gene expression profiles. Neoplasia.

[29] Arredouani MS, Lu B, Bhasin M, Eljanne M, Yue W, Mosquera JM, Bubley GJ, Li V, Rubin MA, Libermann TA, Sanda MG (2009). Identification of the transcription factor single-minded homologue 2 as a potential biomarker and immunotherapy target in prostate cancer. Clin Cancer Res.

[30] Grasso CS, Wu YM, Robinson DR, Cao X, Dhanasekaran SM, Khan AP, Quist MJ, Jing X, Lonigro RJ, Brenner JC, Asangani IA, Ateeq B, Chun SY (2012). The mutational landscape of lethal castration-resistant prostate cancer. Nature.

[31] Holzbeierlein J, Lal P, LaTulippe E, Smith A, Satagopan J, Zhang L, Ryan C, Smith S, Scher H, Scardino P, Reuter V, Gerald WL (2004). Gene expression analysis of human prostate carcinoma during hormonal therapy identifies androgen-responsive genes and mechanisms of therapy resistance. Am J Pathol.

[32] Lapointe J, Li C, Higgins JP, van de Rijn M, Bair E, Montgomery K, Ferrari M, Egevad L, Rayford W, Bergerheim U, Ekman P, DeMarzo AM, Tibshirani R (2004). Gene expression profiling identifies clinically relevant subtypes of prostate cancer. Proc Natl Acad Sci USA.

[33] LaTulippe E, Satagopan J, Smith A, Scher H, Scardino P, Reuter V, Gerald WL (2002). Comprehensive gene expression analysis of prostate cancer reveals distinct transcriptional programs associated with metastatic disease. Cancer Res.

[34] Liu P, Ramachandran S, Ali Seyed M, Scharer CD, Laycock N, Dalton WB, Williams H, Karanam S, Datta MW, Jaye DL, Moreno CS (2006). Sex-determining region Y box 4 is a transforming oncogene in human prostate cancer cells. Cancer Res.

[35] Luo JH, Yu YP, Cieply K, Lin F, Deflavia P, Dhir R, Finkelstein S, Michalopoulos G, Becich M (2002). Gene expression analysis of prostate cancers. Mol Carcinog.

[36] Magee JA, Araki T, Patil S, Ehrig T, True L, Humphrey PA, Catalona WJ, Watson MA, Milbrandt J (2001). Expression profiling reveals hepsin overexpression in prostate cancer. Cancer Res.

[37] Singh D, Febbo PG, Ross K, Jackson DG, Manola J, Ladd C, Tamayo P, Renshaw AA, D’Amico AV, Richie JP, Lander ES, Loda M, Kantoff PW (2002). Gene expression correlates of clinical prostate cancer behavior. Cancer Cell.

[38] Taylor BS, Schultz N, Hieronymus H, Gopalan A, Xiao Y, Carver BS, Arora VK, Kaushik P, Cerami E, Reva B, Antipin Y, Mitsiades N, Landers T (2010). Integrative genomic profiling of human prostate cancer. Cancer Cell.

[39] Tomlins SA, Mehra R, Rhodes DR, Cao X, Wang L, Dhanasekaran SM, Kalyana-Sundaram S, Wei JT, Rubin MA, Pienta KJ, Shah RB, Chinnaiyan AM (2007). Integrative molecular concept modeling of prostate cancer progression. Nat Genet.

[40] Vanaja DK, Cheville JC, Iturria SJ, Young CY (2003). Transcriptional silencing of zinc finger protein 185 identified by expression profiling is associated with prostate cancer progression. Cancer Res.

[41] Varambally S, Yu J, Laxman B, Rhodes DR, Mehra R, Tomlins SA, Shah RB, Chandran U, Monzon FA, Becich MJ, Wei JT, Pienta KJ, Ghosh D (2005). Integrative genomic and proteomic analysis of prostate cancer reveals signatures of metastatic progression. Cancer Cell.

[42] Wallace TA, Prueitt RL, Yi M, Howe TM, Gillespie JW, Yfantis HG, Stephens RM, Caporaso NE, Loffredo CA, Ambs S (2008). Tumor immunobiological differences in prostate cancer between African-American and European-American men. Cancer Res.

[43] Welsh JB, Sapinoso LM, Su AI, Kern SG, Wang-Rodriguez J, Moskaluk CA, Frierson HF, Hampton GM (2001). Analysis of gene expression identifies candidate markers and pharmacological targets in prostate cancer. Cancer Res.

[44] Yu YP, Landsittel D, Jing L, Nelson J, Ren B, Liu L, McDonald C, Thomas R, Dhir R, Finkelstein S, Michalopoulos G, Becich M, Luo JH (2004). Gene expression alterations in prostate cancer predicting tumor aggression and preceding development of malignancy. J Clin Oncol.

[45] Zhang W, Chen T, Wan T, He L, Li N, Yuan Z, Cao X (2000). Cloning of DPK, a novel dendritic cell-derived protein kinase activating the ERK1/ERK2 and JNK/SAPK pathways. Biochem Biophys Res Commun.

[46] Chen Z, Raman M, Chen L, Lee SF, Gilman AG, Cobb MH (2003). TAO (thousand-and-one amino acid) protein kinases mediate signaling from carbachol to p38 mitogen-activated protein kinase and ternary complex factors. J Biol Chem.

[47] Romanuik TL, Wang G, Holt RA, Jones SJ, Marra MA, Sadar MD (2009). Identification of novel androgen-responsive genes by sequencing of LongSAGE libraries. BMC Genomics.

[48] Keng VW, Villanueva A, Chiang DY, Dupuy AJ, Ryan BJ, Matise I, Silverstein KA, Sarver A, Starr TK, Akagi K, Tessarollo L, Collier LS, Powers S (2009). A conditional transposon-based insertional mutagenesis screen for genes associated with mouse hepatocellular carcinoma. Nat Biotechnol.

[49] Triller N, Korosec P, Kern I, Kosnik M, Debeljak A (2006). Multidrug resistance in small cell lung cancer: expression of P-glycoprotein, multidrug resistance protein 1 and lung resistance protein in chemo-naive patients and in relapsed disease. Lung Cancer.

[50] Jin F, Zhao L, Guo YJ, Zhao WJ, Zhang H, Wang HT, Shao T, Zhang SL, Wei YJ, Feng J, Jiang XB, Zhao HY (2010). Influence of Etoposide on anti-apoptotic and multidrug resistance-associated protein genes in CD133 positive U251 glioblastoma stem-like cells. Brain Res.

[51] Vesuna F, Lisok A, Kimble B, Raman V (2009). Twist modulates breast cancer stem cells by transcriptional regulation of CD24 expression. Neoplasia.

[52] Zhu Z, Hao X, Yan M, Yao M, Ge C, Gu J, Li J (2010). Cancer stem/progenitor cells are highly enriched in CD133+CD44+ population in hepatocellular carcinoma. Int J Cancer.

[53] Liu C, Li Z, Bi L, Li K, Zhou B, Xu C, Huang J, Xu K (2014). NOTCH1 signaling promotes chemoresistance via regulating ABCC1 expression in prostate cancer stem cells. Mol Cell Biochem.

[54] Paik S, Shak S, Tang G, Kim C, Baker J, Cronin M, Baehner FL, Walker MG, Watson D, Park T, Hiller W, Fisher ER, Wickerham DL (2004). A multigene assay to predict recurrence of tamoxifen-treated, node-negative breast cancer. N Engl J Med.

[55] Trotman L, Powers S (2010). New views into the prostate cancer genome. Cancer Cell.

[56] Basil CF, Zhao Y, Zavaglia K, Jin P, Panelli MC, Voiculescu S, Mandruzzato S, Lee HM, Seliger B, Freedman RS, Taylor PR, Hu N, Zanovello P (2006). Common cancer biomarkers. Cancer Res.

[57] Cerami E, Gao J, Dogrusoz U, Gross BE, Sumer SO, Aksoy BA, Jacobsen A, Byrne CJ, Heuer ML, Larsson E, Antipin Y, Reva B, Goldberg AP (2012). The cBio cancer genomics portal: an open platform for exploring multidimensional cancer genomics data. Cancer Discov.

[58] Network CG, and Cancer Genome Atlas Research Network (2015). The Molecular Taxonomy of Primary Prostate Cancer. Cell.

[59] Aguirre-Gamboa R, Gomez-Rueda H, Martinez-Ledesma E, Martinez-Torteya A, Chacolla-Huaringa R, Rodriguez-Barrientos A, Tamez-Pena JG, Trevino V (2013). SurvExpress: an online biomarker validation tool and database for cancer gene expression data using survival analysis. PLoS One.

[60] Sharifi N, Gulley JL, Dahut WL (2005). Androgen deprivation therapy for prostate cancer. JAMA.

[61] Feldman BJ, Feldman D (2001). The development of androgen-independent prostate cancer. Nat Rev Cancer.

[62] Taplin ME, Bubley GJ, Shuster TD, Frantz ME, Spooner AE, Ogata GK, Keer HN, Balk SP (1995). Mutation of the androgen-receptor gene in metastatic androgen-independent prostate cancer. N Engl J Med.

[63] Lawrence MS, Stojanov P, Polak P, Kryukov GV, Cibulskis K, Sivachenko A, Carter SL, Stewart C, Mermel CH, Roberts SA, Kiezun A, Hammerman PS, McKenna A (2013). Mutational heterogeneity in cancer and the search for new cancer-associated genes. Nature.

[64] Zhang J, Kobert K, Flouri T, Stamatakis A (2014). PEAR: a fast and accurate Illumina Paired-End reAd mergeR. Bioinformatics.

[65] Kent WJ (2002). BLAT--the BLAST-like alignment tool. Genome Res.

[66] Trobridge GD, Miller DG, Jacobs MA, Allen JM, Kiem HP, Kaul R, Russell DW (2006). Foamy virus vector integration sites in normal human cells. Proc Natl Acad Sci USA.

[67] Gao J, Aksoy BA, Dogrusoz U, Dresdner G, Gross B, Sumer SO, Sun Y, Jacobsen A, Sinha R, Larsson E, Cerami E, Sander C, Schultz N (2013). Integrative analysis of complex cancer genomics and clinical profiles using the cBioPortal. Sci Signal.

