# Supplementary material for: Replication-incompetent gammaretroviral and lentiviral vector-based insertional mutagenesis screens identify prostate cancer progression genes
Source: Oncotarget. 2018 Feb 15;9(21):15451–63. doi: 10.18632/oncotarget.24503 (PMC5884640; doi:10.18632/oncotarget.24503)
Supplement: Supplementary file 1 [file oncotarget-09-15451-s001.pdf]

Replication-incompetent gammaretroviral and lentiviral vector-based insertional mutagenesis screens identify prostate cancer progression genes

SUPPLEMENTARY MATERIALS

γRV vector proviral integration site near *OR2A14* gene on chromosome 7

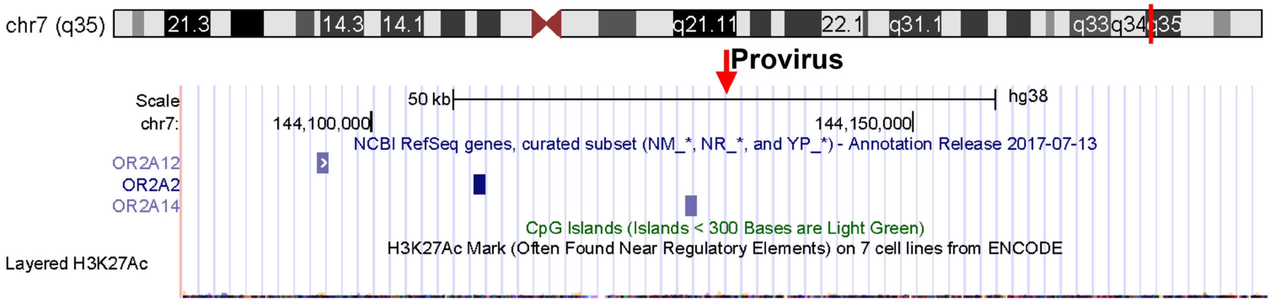

γRV vector proviral integration site near *FER1L6* gene on chromosome 8

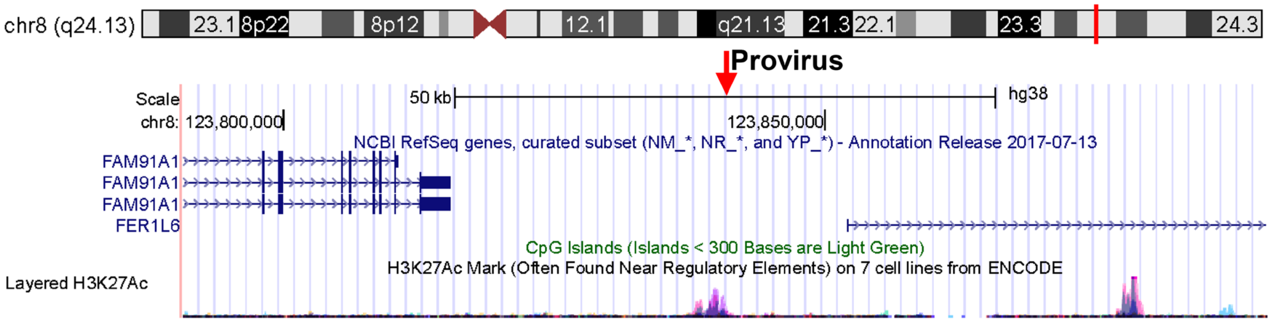

γRV vector proviral integration site in *TAOK3* gene on chromosome 12

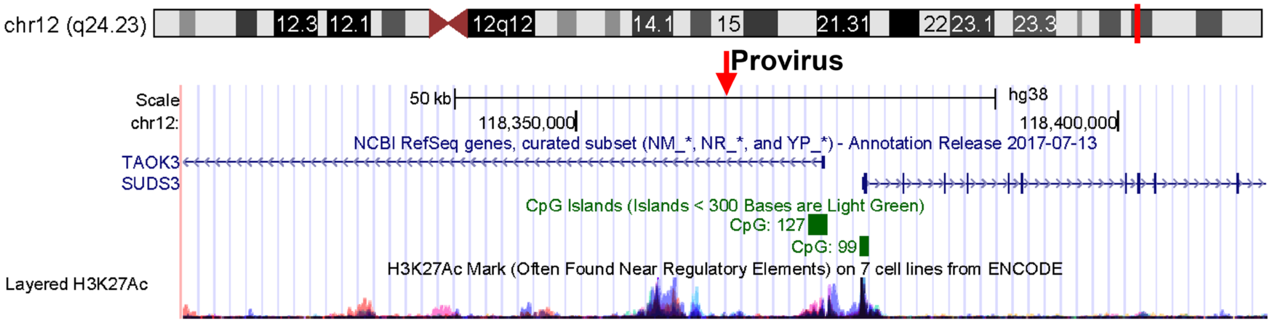

LV vector proviral integration site in *MAN1A2* gene on chromosome 1

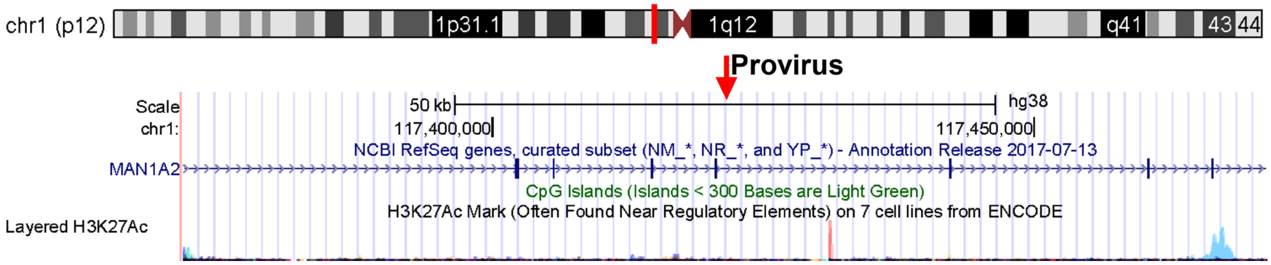

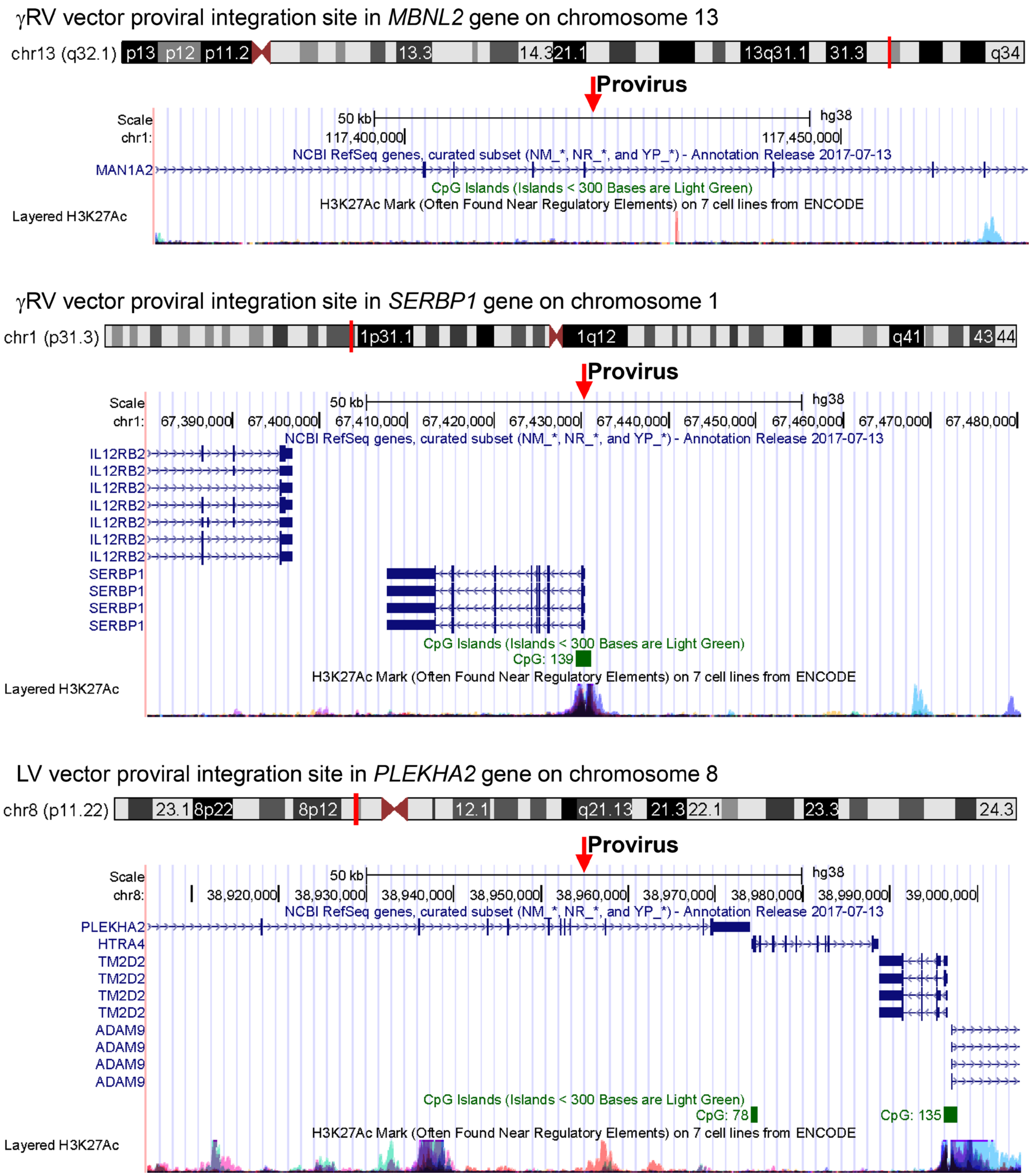
γRV vector proviral integration site in MBNL2 gene on chromosome 13
chr13 (q32.1)
p13
p12
p11.2
13.3
14.3
21.1
13q31.1
31.3
q34
Provirus
Scale
50 kb
hg38
chr1:
117,400,000
117,450,000
NCBI RefSeq genes, curated subset (NM_*, NR_*, and YP_*) - Annotation Release 2017-07-13
MAN1A2
CpG Islands (Islands < 300 Bases are Light Green)
H3K27Ac Mark (Often Found Near Regulatory Elements) on 7 cell lines from ENCODE
Layered H3K27Ac
γRV vector proviral integration site in SERBP1 gene on chromosome 1
chr1 (p31.3)
1p31.1
1q12
q41
43
44
Provirus
Scale
50 kb
hg38
chr1:
67,390,000
67,400,000
67,410,000
67,420,000
67,430,000
67,440,000
67,450,000
67,460,000
67,470,000
67,480,000
NCBI RefSeq genes, curated subset (NM_*, NR_*, and YP_*) - Annotation Release 2017-07-13
IL12RB2
IL12RB2
IL12RB2
IL12RB2
IL12RB2
IL12RB2
IL12RB2
SERBP1
SERBP1
SERBP1
SERBP1
CpG Islands (Islands < 300 Bases are Light Green)
CpG: 139
H3K27Ac Mark (Often Found Near Regulatory Elements) on 7 cell lines from ENCODE
Layered H3K27Ac
LV vector proviral integration site in PLEKHA2 gene on chromosome 8
chr8 (p11.22)
23.1
8p22
8p12
12.1
q21.13
21.3
22.1
23.3
24.3
Provirus
Scale
50 kb
hg38
chr8:
38,920,000
38,930,000
38,940,000
38,950,000
38,960,000
38,970,000
38,980,000
38,990,000
39,000,000
NCBI RefSeq genes, curated subset (NM_*, NR_*, and YP_*) - Annotation Release 2017-07-13
PLEKHA2
HTRA4
TM2D2
TM2D2
TM2D2
TM2D2
ADAM9
ADAM9
ADAM9
ADAM9
CpG Islands (Islands < 300 Bases are Light Green)
CpG: 78
CpG: 135
H3K27Ac Mark (Often Found Near Regulatory Elements) on 7 cell lines from ENCODE
Layered H3K27Ac

LV vector proviral integration site in *SPTAN1* gene on chromosome 9

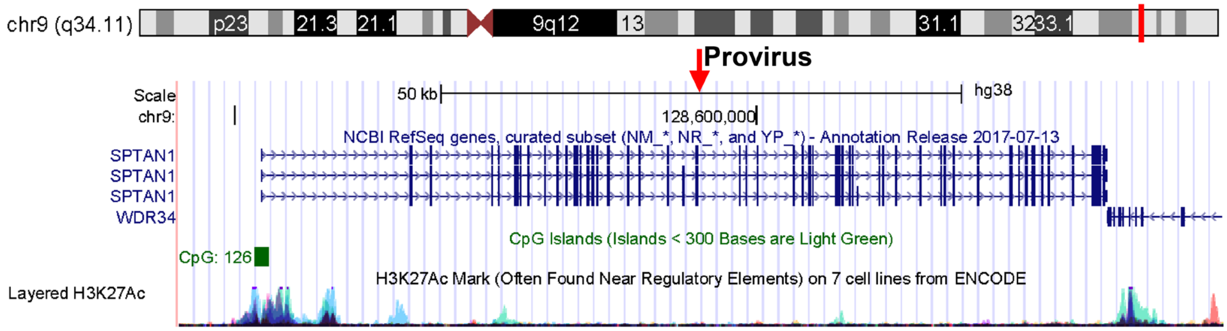

LV vector proviral integration site near *ADAMTS1* gene on chromosome 21

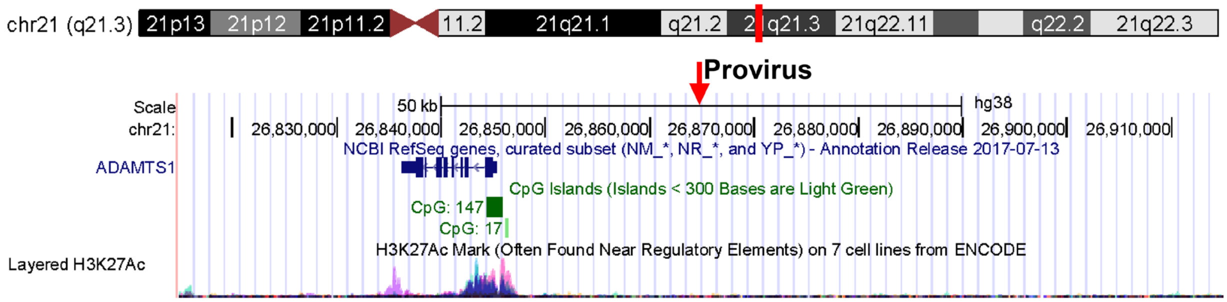

LV vector proviral integration site near *SLC30A5* gene on chromosome 5

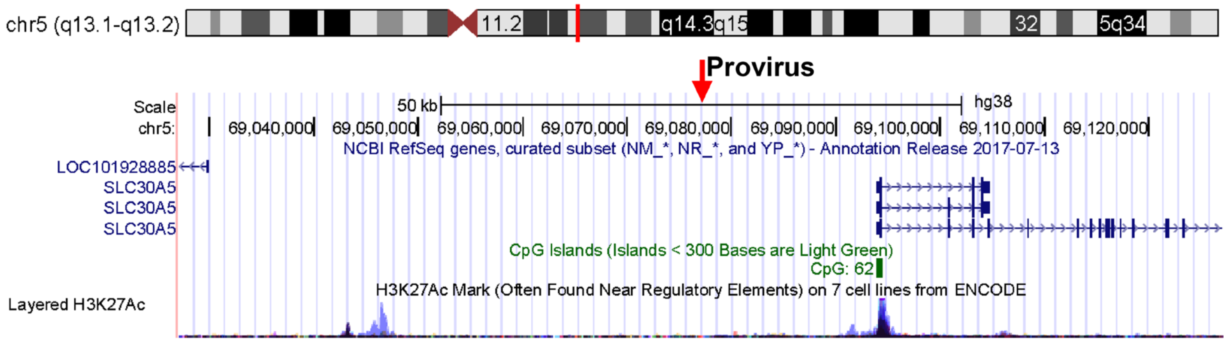

LV vector proviral integration site in *ABCC1* gene on chromosome 16

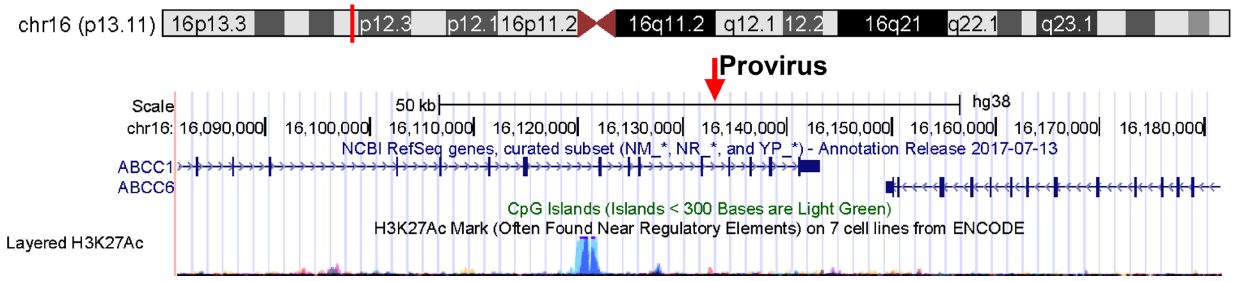

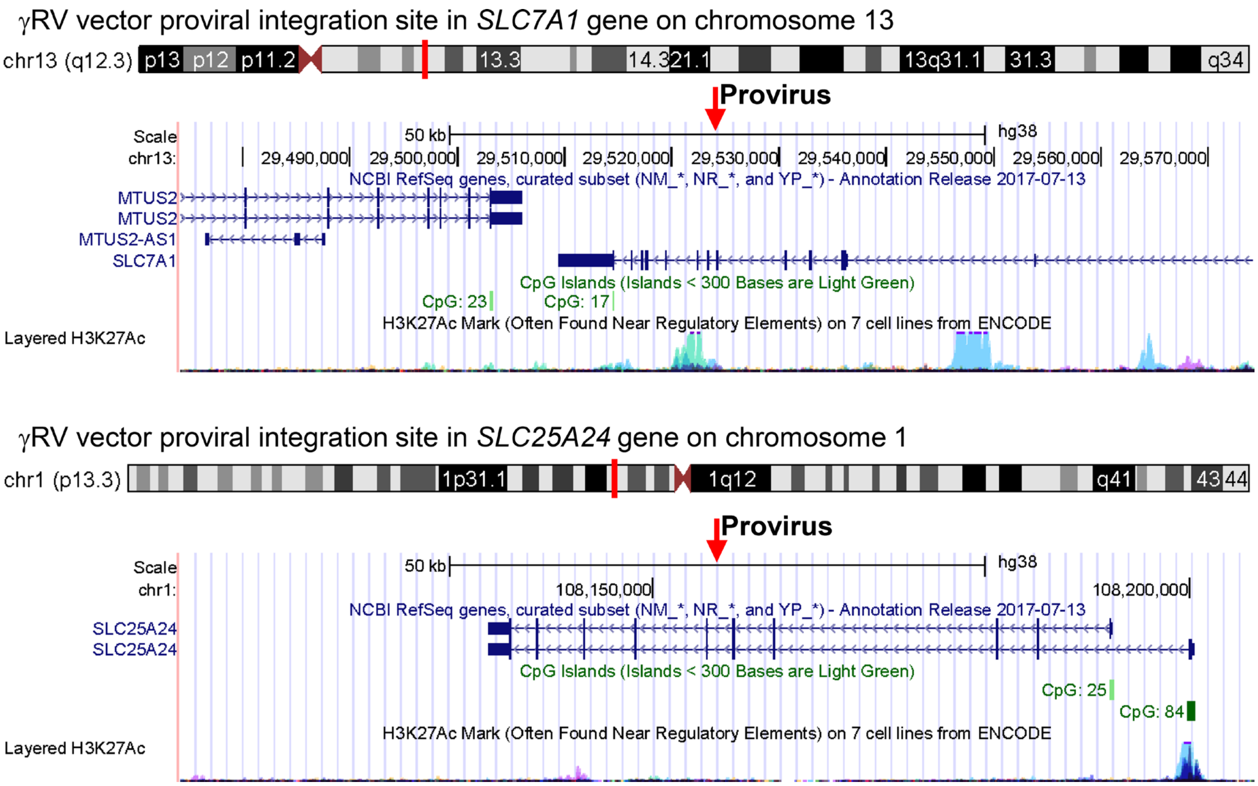

Supplementary Figure 1: Proviral integration sites mapped to the human genome(hg38) using the UCSC genome browser. Redarrows indicate the provirus integration site.

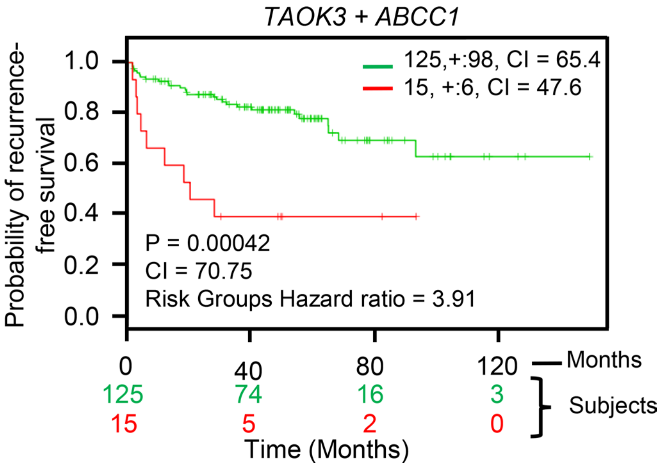

Supplementary Figure 2: Combination of TAOK3 and ABCC1 expression predicts clinical outcomes in AIPC patients after treatment. The Kaplan-Meier survival curves generated using SurvExpress biomarker tool showing the ability of a γRV and LV-tagged gene expression to predict recurrence-free survival outcome in PC patients after androgendeprivation therapy usingcohorts fromTayloretal. 2010 data.The insets intoprightre presents number of individuals, number censored, and concordance index (CI) of each riskgroupsand '+' representcensoring samples. Highand lowrisk group sare show ninred and green respectively. Box-plots show expression levels and *p*-values resulting from *t*-test of the differenc eexpression between high risk (red) andl ow risk (green) groupsin BC patients.

1. Prostate Carcinoma vs. Normal. *Arredouani Prostate, Clin Cancer Res, 2009*
2. Prostate Carcinoma vs. Normal. *Grasso Prostate, Nature, 2012*
3. Prostate Carcinoma vs. Normal. *Holzbeierlein Prostate, Am J Pathol, 2004*
4. Prostate Carcinoma vs. Normal. *Lapointe Prostate, Proc Natl Acad Sci U S A, 2004*
5. Prostate Carcinoma vs. Normal. *LaTulippe Prostate, Cancer Res, 2002*
6. Prostate Carcinoma vs. Normal. *Liu Prostate, Cancer Res, 2006*
7. Prostate Carcinoma vs. Normal. *Luo Prostate 2, Mol Carcinog, 2002*
8. Prostate Carcinoma vs. Normal. *Magee Prostate, Cancer Res, 2001*
9. Prostate Carcinoma vs. Normal. *Singh Prostate, Cancer Cell, 2002*
10. Prostate Carcinoma vs. Normal. *Taylor Prostate 3, Cancer Cell, 2010*
11. Acinar Prostate Adenocarcinoma vs. Normal. *TCGA Prostate, No Associated Paper, 2012*
12. Prostate Adenocarcinoma vs. Normal. *TCGA Prostate, No Associated Paper, 2012*
13. Benign Prostatic Hyperplasia Epithelia vs. Normal. *Tomlins Prostate, Nat Genet, 2007*
14. Benign Prostatic Hyperplasia Stroma vs. Normal. *Tomlins Prostate, Nat Genet, 2007*
15. Prostate Carcinoma Epithelia vs. Normal. *Tomlins Prostate, Nat Genet, 2007*
16. Prostatic Intraepithelial Neoplasia Epithelia vs. Normal. *Tomlins Prostate, Nat Genet, 2007*
17. Prostate Adenocarcinoma vs. Normal. *Vanaja Prostate, Cancer Res, 2003*
18. Prostate Carcinoma vs. Normal. *Varambally Prostate, Cancer Cell, 2005*
19. Prostate Adenocarcinoma vs. Normal. *Wallace Prostate, Cancer Res, 2008*
20. Prostate Carcinoma vs. Normal. *Welsh Prostate, Cancer Res, 2001*
21. Prostate Carcinoma vs. Normal. *Yu Prostate, J Clin Oncol, 2004*

Supplementary Figure 3: Datasets used for Oncomine analysis.

Supplementary Table 1: Provirus tagged candidate PC AQ2 metastasis genes. See Supplementary_Table_1

REFERENCES

1. Arredouani MS, Lu B, Bhasin M, Eljanne M, Yue W, Mosquera JM, Bubley GJ, Li V, Rubin MA, Libermann TA, Sanda MG. Identification of the transcription factor single-minded homologue 2 as a potential biomarker and immunotherapy target in prostate cancer. Clin Cancer Res. 2009; 15:5794–802. https://doi.org/10.1158/1078-0432.CCR-09-0911.
2. Grasso CS, Wu YM, Robinson DR, Cao X, Dhanasekaran SM, Khan AP, Quist MJ, Jing X, Lonigro RJ, Brenner JC, Asangani IA, Ateeq B, Chun SY, et al. The mutational landscape of lethal castration-resistant prostate cancer. Nature. 2012; 487:239–43. https://doi.org/10.1038/nature11125.
3. Holzbeierlein J, Lal P, LaTulippe E, Smith A, Satagopan J, Zhang L, Ryan C, Smith S, Scher H, Scardino P, Reuter V, Gerald WL. Gene expression analysis of human prostate carcinoma during hormonal therapy identifies androgen-responsive genes and mechanisms of therapy resistance. Am J Pathol. 2004; 164:217–27. https://doi.org/10.1016/S0002-9440(10)63112-4.
4. Lapointe J, Li C, Higgins JP, van de Rijn M, Bair E, Montgomery K, Ferrari M, Egevad L, Rayford W, Bergerheim U, Ekman P, DeMarzo AM, Tibshirani R, et al. Gene expression profiling identifies clinically relevant subtypes of prostate cancer. Proc Natl Acad Sci USA. 2004; 101:811–16. https://doi.org/10.1073/pnas.0304146101.
5. LaTulippe E, Satagopan J, Smith A, Scher H, Scardino P, Reuter V, Gerald WL. Comprehensive gene expression analysis of prostate cancer reveals distinct transcriptional programs associated with metastatic disease. Cancer Res. 2002; 62:4499–506.
6. Liu P, Ramachandran S, Ali Seyed M, Scharer CD, Laycock N, Dalton WB, Williams H, Karanam S, Datta MW, Jaye DL, Moreno CS. Sex-determining region Y box 4 is a transforming oncogene in human prostate cancer cells. Cancer Res. 2006; 66:4011–19. https://doi.org/10.1158/0008-5472.CAN-05-3055.
7. Luo JH, Yu YP, Cieply K, Lin F, Deflavia P, Dhir R, Finkelstein S, Michalopoulos G, Becich M. Gene expression analysis of prostate cancers. Mol Carcinog. 2002; 33:25–35. https://doi.org/10.1002/mc.10018.
8. Magee JA, Araki T, Patil S, Ehrig T, True L, Humphrey PA, Catalona WJ, Watson MA, Milbrandt J. Expression profiling reveals hepsin overexpression in prostate cancer. Cancer Res. 2001; 61:5692–96.
9. Singh D, Febbo PG, Ross K, Jackson DG, Manola J, Ladd C, Tamayo P, Renshaw AA, D'Amico AV, Richie JP, Lander ES, Loda M, Kantoff PW, et al. Gene expression correlates of clinical prostate cancer behavior. Cancer Cell. 2002; 1:203–09. https://doi.org/10.1016/S1535-6108(02)00030-2.
10. Taylor BS, Schultz N, Hieronymus H, Gopalan A, Xiao Y, Carver BS, Arora VK, Kaushik P, Cerami E, Reva B, Antipin Y, Mitsiades N, Landers T, et al. Integrative genomic profiling of human prostate cancer. Cancer Cell. 2010; 18:11–22. https://doi.org/10.1016/j.ccr.2010.05.026.
11. Tomlins SA, Mehra R, Rhodes DR, Cao X, Wang L, Dhanasekaran SM, Kalyana-Sundaram S, Wei JT, Rubin MA, Pienta KJ, Shah RB, Chinnaiyan AM. Integrative molecular concept modeling of prostate cancer progression. Nat Genet. 2007; 39:41–51. https://doi.org/10.1038/ng1935.
12. Vanaja DK, Cheville JC, Iturria SJ, Young CY. Transcriptional silencing of zinc finger protein 185 identified by expression profiling is associated with prostate cancer progression. Cancer Res. 2003; 63:3877–82.
13. Varambally S, Yu J, Laxman B, Rhodes DR, Mehra R, Tomlins SA, Shah RB, Chandran U, Monzon FA, Becich MJ, Wei JT, Pienta KJ, Ghosh D, et al. Integrative genomic and proteomic analysis of prostate cancer reveals signatures of metastatic progression. Cancer Cell. 2005; 8:393–406. https://doi.org/10.1016/j.ccr.2005.10.001.
14. Wallace TA, Prueitt RL, Yi M, Howe TM, Gillespie JW, Yfantis HG, Stephens RM, Caporaso NE, Loffredo CA, Ambs S. Tumor immunobiological differences in prostate cancer between African-American and European-American men. Cancer Res. 2008; 68:927–36. https://doi.org/10.1158/0008-5472.CAN-07-2608.
15. Welsh JB, Sapinoso LM, Su AI, Kern SG, Wang-Rodriguez J, Moskaluk CA, Frierson HF Jr, Hampton GM. Analysis of gene expression identifies candidate markers and pharmacological targets in prostate cancer. Cancer Res. 2001; 61:5974–78.
16. Yu YP, Landsittel D, Jing L, Nelson J, Ren B, Liu L, McDonald C, Thomas R, Dhir R, Finkelstein S, Michalopoulos G, Becich M, Luo JH. Gene expression alterations in prostate cancer predicting tumor aggression and preceding development of malignancy. J Clin Oncol. 2004; 22:2790–99. https://doi.org/10.1200/JCO.2004.05.158.
